# A New Microfluidic Platform for Studying Natural Killer Cell and Dendritic Cell Interactions

**DOI:** 10.3390/mi10120851

**Published:** 2019-12-05

**Authors:** Jolly Hipolito, Hagit Peretz-Soroka, Manli Zhang, Ke Yang, Soheila Karimi-Abdolrezaee, Francis Lin, Sam K.P. Kung

**Affiliations:** 1 Department of Immunology, University of Manitoba, 417 Apotex Centre, 750 McDermot Avenue, Winnipeg, MB R3E 0T5, Canada; Jolly.Hipolito@umanitoba.ca (J.H.); Man.Zhang@umanitoba.ca (M.Z.); 2 Department of Physics and Astronomy, University of Manitoba, Winnipeg, MB R3T 2N2, Canada; hagit.peretz@gmail.com (H.P.-S.); keyang@rntek.cas.cn (K.Y.); 3 Department of Physiology and Pathophysiology, University of Manitoba, Winnipeg, MB R3E 0T5, Canada; Soheila.Karimi@umanitoba.ca; 4 Department of Biosystems Engineering, University of Manitoba, Winnipeg, MB R3T 2N2, Canada

**Keywords:** natural killer, dendritic, migration, interaction, crosstalk, microfluidic device

## Abstract

The importance of the bi-directional natural killer–dendritic cell crosstalk in coordinating anti-tumour and anti-microbial responses in vivo has been well established. However, physical parameters associated with natural killer–dendritic cell interactions have not been fully elucidated. We have previously used a simple “Y” shaped microfluidic device to study natural killer cell-migratory responses toward chemical gradients from a conditioned medium of dendritic cells. There are, however, limitations of the Y-shaped microfluidic devices that could not support higher throughput analyses and studies of cell–cell interactions. Here, we report two novel microfluidic devices (D^3^-Chip, T^2^-Chip) we applied in advanced studies of natural killer-cell migrations and their interactions with dendritic cells in vitro. The D^3^-Chip is an improved version of the previously published Y-shaped device that supports high-throughput analyses and docking of the cells of interest in the migration assay before they are exposed to a chemical gradient. The T^2^-Chip is created to support analyses of natural killer–dendritic cell cell–cell interactions without the requirement of promoting a natural killer cell to migrate long distances to find a loaded dendritic cell in the device. Using these two microfluidic platforms, we observe quantitative differences in the abilities of the immature and lipopolysaccharide-activated mature dendritic cells to interact with activated natural killer cells. The contact time between the activated natural killer cells and immature dendritic cells is significantly longer than that of the mature dendritic cells. There is a significantly higher frequency of an immature dendritic cell coming into contact with multiple natural killer cells and/or making multiple simultaneous contacts with multiple natural killer cells. To contrast, an activated natural killer cell has a significantly higher frequency of coming into contact with the mature dendritic cells than immature dendritic cells. Collectively, these differences in natural killer–dendritic cell interactions may underlie the differential maturation of immature dendritic cells by activated natural killer cells. Further applications of these microfluidic devices in studying natural killer–dendritic cell crosstalk under defined microenvironments shall enrich our understanding of the functional regulations of natural killer cells and dendritic cells in the natural killer–dendritic cell crosstalk.

## 1. Introduction

Regulation of immune cell migrations to proper microenvironments is critical for development of specific cell types and/or induction of specific immune responses [1]. Classical natural killer (NK) cells are granulocytic lymphocytes that were first identified because of their “natural” ability to eliminate “abnormal” transformed cells without prior sensitization [2]. NK cells acquire specific chemokine and non-chemokine surface receptors (ranging from CXC, CC, C and CX3C motif chemokine receptors) to regulate migrations into peripheral organs or inflamed lymph nodes to facilitate immune-surveillance [3,4,5,6,7,8]. Additionally, NK cells interact with dendritic cells (DC), a critical cell type that regulates adaptive immunity [8,9]. DC produce a number of chemokines (such as CXCL8, CXCL9, IP-10, CXCL11) to induce NK-cell migrations [10,11]. The importance of the bi-directional NK–DC crosstalk in coordinating anti-tumour and anti-microbial responses in vivo has been well established [12]. However, physical parameters associated with NK–DC interactions have not been fully elucidated.

Microfluidic-based migration systems offer the advantages of better control and manipulation of the fluidic behaviour in microfluidic devices [13,14]. Our laboratories have previously used a simple “Y” shaped microfluidic device to demonstrate directed NK-cell migrations in controlled gradient environments created by the conditioned medium from the activated DC, and quantitative cell-migration data analysis (chemotaxis, chemo-repulsion, speed, angular migration) at the single-cell level [15]. Key features of the Y-shaped device are: simple design, relatively good control of stable chemical gradients (single or co-existing), and the ability to trace single cell migration in real-time. There are, however, limitations of the Y-shaped microfluidic devices. It includes the requirement of external syringe pumps for chemical flow infusion, relatively low throughput, and the difficulty in configuring different cell types together in the device to facilitate cell–cell interaction studies. Additionally, the inability to control the initial start positions of cells on a defined gradient profile result in NK cells having a slightly different chemical concentration at their initial positions. The latter might affect analyses of cell migrations in weak chemotactic conditions.

Here, we report our recent technical development of two novel microfluidic devices (D^3^-Chip, T^2^-Chip) to further advance studies of NK-cell migrations and their interactions with dendritic cells in vitro.

## 2. Materials and Methods

### 2.1. Natural Killer (NK) Cell Isolation and Activation

Animal work was approved by the University of Manitoba’s Review Board. C57BL/6 and the “CAG-EYFP” Transgenic mice that express enhanced yellow fluorescent protein (EYFP) under the CAG promoter [16] were obtained from Animal Care Services at the University of Manitoba. NK cells were isolated from the spleens using an EasySep^TM^ Mouse NK Negative Selection Kit (Stemcell Technologies, Vancouver, BC, Canada), as described [15]. Isolated NK cells were incubated in mouse medium consisting of RPMI-1640 (HyClone, Thermo Fisher Scientific, Rockford, IL, USA) with 10% fetal bovine serum (HyClone), 1% penicillin/streptomycin/-Glutamine (Invitrogen), 50 μM 2-mercaptoethanol, and 1000 units/mL recombinant human IL-2 (Peprotech, Rocky Hill, NJ, USA) in an incubator set at 37 °C with 5% CO_2_ injection for 4 days.

### 2.2. Preparation of Dendritic Cell (DC) Cultures

Bone marrow (BM) derived DC were cultured as described previously [15]. Briefly, mouse BM cells from the femur and tibiae are plated at 1 × 10^6^ cells/mL in RPMI-1640 culture medium containing 10% fetal bovine serum, 1% penicillin/streptomycin/L-Glutamine (Invitrogen), 2-ME, and 20 ng/mL GM-CSF (Peprotech, Rocky Hill, NJ, USA). Immature DC was obtained on day 7. To mature these immature DC further, lipopolysaccharide (LPS, Sigma–Aldrich, Oakville, ON, Canada) at 1 μg/mL was added to the DC culture medium on day 7 for another 24-hours. Supernatant of the immature DC and mature DC (conditioned medium) was collected on day 8 and centrifuged at 1500 RPM for 10 minutes to remove cellular debris. They were used to generate a gradient for chemotaxis assays in experiments.

### 2.3. Microfluidic Device Preparation

Standard photolithography and soft-lithography methods were followed for microfluidic device fabrication [17]. Microfluidic designs were completed using the Solidworks software (Version 2013, Dassault Systems, Providence, RI, USA). Photomasks of completed microfluidic designs were printed onto transparency films, at a 24,000 dpi resolution (Fineline Imaging, Colorado Springs, CO, USA). SU-8 photoresist was spin-coated onto silicon wafers (MicroChem Corporation, Westborough, MA, USA), and then ultraviolet light was projected through the photomask to the silicon wafers [17]. D^3^-Chip devices were designed with a height and width of 70 μm and 220 μm, respectively, while the T^2^-Chip devices had a height of 70 µm and a width to the tips of the traps and base of the traps of 200 µm and 255 µm, respectively. Additionally, a ~3μm high ceiling for the docking structure was constructed to align and trap cells for both devices. Polydimethylsiloxane replicas were constructed using the finished silicone master devices. All inlets, outlets and cell loading wells were drilled through the polydimethylsiloxane (PDMS) block. An air plasma cleaner (Harrick Plasma, Ithaca, NY, USA) was used to adhere PDMS replicas to microscope glass slides. De-ionized water was injected into the wells to maintain the hydrophilic nature of the device for storage and later use. Microfluidic devices were coated with 31.25 μg/mL fibronectin for 1 hour in an incubator set at 37 °C with a 5% CO_2_ injection, followed by 0.4% bovine serum albumin (BSA) in RPMI-1640 blocking for another 1 hour at room temperature.

### 2.4. Cell Migration and Cell–Cell Interaction Experiments

Regarding cell migration experiments, NK cells were seeded through cell loading inlets. They were trapped and aligned accordingly, relative to the gradient. Different chemoattractant solutions were used alongside mouse medium in each source well, creating a concentration gradient. The pressure difference between the source wells and outlets allowed the flow of chemoattractant and mouse medium through the main gradient channel, generating a chemoattractant gradient. An inverted Nikon Ti-U microscope was used to capture differential interference contrast (DIC) time-lapse images at six frames per minute for a total of 40 minutes using a 10× objective lens. Fluorescein isothiocyanate (FITC)-Dextran was added to a chemoattractant solution to visually define gradient profiles. Cell–cell interaction experiments were done by DIC time-lapse images in an inverted Nikon Ti-U microscope at six frames per minute for a total of 40 minutes with a 40× objective lens. NK cells were seeded through the cell loading inlets of the D^3^-Chip while immature DC or mature DC cells were seeded and randomly adhered in the main gradient channel. Similarly, chemoattractant solutions were added alongside mouse medium in each source well, creating a concentration gradient. While studying NK–DC interactions using the T^2^-Chip devices, NK cells were seeded similarly in the cell loading port while DC were loaded directly into the “trap” (small compartment structures) in the main gradient channel. Migrations of NK cells into the traps through the barrier to interact with the “docked” DC were analyzed.

## 3. Data Analysis

Migration and interaction were recorded by DIC time-lapse microscopy as described above. NK and DCs were manually tracked on the NIH ImageJ software (NIH, Bethesda, MD, USA). Using the “point” or “multi-point” tool on NIH ImageJ, NK cell positions were calculated for their distance travelled in 10-minute intervals for 40 minutes by subtracting the distance at which an NK cell had travelled at its final position to its initial position at the dock. NK cell positions were measured, relative to the docking structure, every 10 minutes up to 40 minutes. Using Excel, the migration distance from the cell to the docking structure was calculated. Cell speed was obtained through analytical software called Chemotaxis Migration and Tools. In vitro cell interaction assays in the D^3^-Chip and T^2^-Chip devices were used to measure the duration of cell contact. Frequency of interaction and simultaneous interaction cell counts were measured on the T^2^-Chip. The Student’s *t*-test was used to calculate a significance of p < 0.05, p < 0.01, and p < 0.001 between conditions. OriginPro 8.5 (OriginLab, Northampton, MA, USA) was used to plot and generate graphs accordingly.

## 4. Results

### 4.1. The Use of the D^3^-Chip Microfluidic Device in the Analyses of Chemotactic Responses of NK-Cells in NK–DC Crosstalk

**Figure 1:**
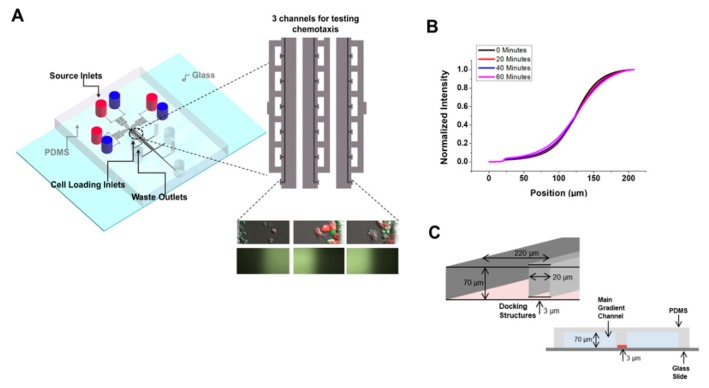
Device schematic and gradient profile calibration of the D^3^-Chip. (**A**) A schematic illustration and gradient profile using FITC-dextran of the D^3^-Chip; (**B**) The gradient profile of the D^3^-Chip for 60 min, indicated by one of the gradient generating channels as a representation; **(C)** Illustration of the cell docking structure. Cells would dock at the barrier and change their morphologies to squeeze and migrate under the ~3 µm gap into the main gradient channel. A 2D cross section view also is visualized to show the docking area and the main gradient area, separated by the docking structure with only the ~3 µm gap to connect the two sections.

The D^3^-Chip device [18] is an improved, high-throughput version of the previously published Y-shaped device (Figure 1). It is comprised of three independent migration assay modules in which each module is comprised of a main gradient channel, cell-docking structure, compensation zone, source, waste and cell-loading wells. Mouse media and DC conditioned media of interest were added to the source wells, generating a concentration gradient via laminar flow. FITC-Dextran was used to test the integrity of the generated gradient throughout the 40 minutes. Three independently controlled microfluidic experiments could be performed in parallel, thus improving the throughput of the migration analyses. Additionally, IL-2 activated NK cells were loaded in the docking structure before the assay. These NK cells, therefore, were trapped and aligned at the same “start” position at the lowest density gradient created in the main gradient channels. IL-2 activated NK cells would then squeeze through the ~3 μm gap and migrate into the main channel and toward the other end where CM concentration was higher. Less IL-2 activated NK cells would pass through the ~3 μm gap of the barrier in the plain medium control. Chemotaxis was measured merely by counting the number of cells that migrated out of the initial position at a specific time point.

**Figure 2:**
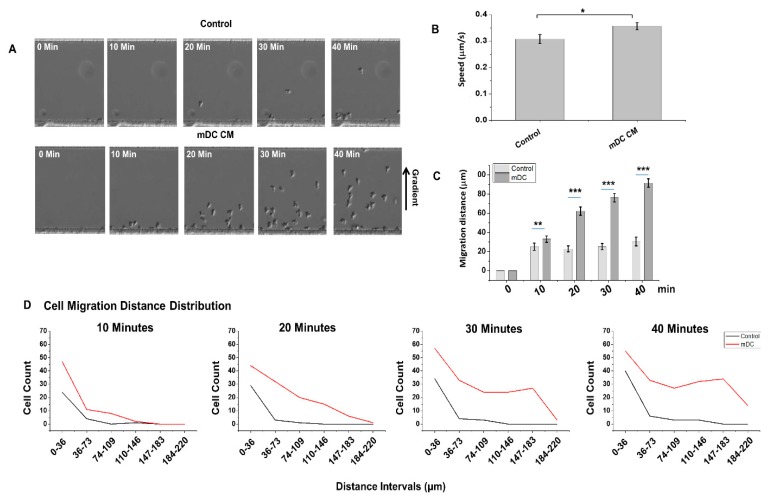
Migrations of IL-2 activated NK cells toward the conditioned medium from the LPS-activated mature DC in the D^3^-Chip. (**A**) Representative cell images of NK cell chemotaxis in a culture medium control, mature DC supernatant gradient, and immature DC supernatant gradient at time 0, 10, 20, 30, and 40 min. Images were corrected on PowerPoint to articulate cell outlines for clear presentation. (**B**) Cell speed and (**C**) migration distance for the culture medium control, mature DC supernatant gradient at time 0, 10, 20, 30, and 40 minutes. The error bar indicates the standard error of the mean (SEM). *, **, and *** indicate *p* < 0.05, *p* < 0.01, and *p* < 0.001, respectively, using the one-way ANOVA test. Data shown are from an average of two experiments. (**D**) A histogram representing an NK cell count distribution at different distances of the D^3^-Chip at time 10, 20, 30 and 40 minutes. Cell counts were organized into six sections from 0–36 μm, 37–73 μm, 74–109 μm, 110–146 μm, 147–183 μm, and 184–220 μm. Data shown are from an average of two repeat experiments.

We used the D^3^-Chip to observe activated NK-cell migrations toward the chemical gradient created by the conditioned medium from LPS-mature DC over 40 min under a microscope (Figure 2, Supplementary Movie S1). A gradient created by plain, complete tissue culture medium was used as a “random” migration control. As shown in Figure 2A, we observed very few NK-cell migrations toward the control medium gradient of plain tissue culture medium within the 40-minute time-lapse we measured (Figure 2A, top panel). This was in contrast to the strong and significant chemotaxis of NK cells we observed under the gradient created by the LPS-matured DC induced (Figure 2A, lower panel). These results were consistent with the migratory responses we reported using the Y-shaped devices previously [15]. Further tracking of these migrating cells allowed us to measure speed (Figure 2B), migration distance (Figure 2C) and migrating cell count distribution at different distances of the D^3^-chip (Figure 2D). We observed that NK-cell migration speed (Figure 2B), migration distance (Figure 2C) and the distribution of NK-cell migration from 0 to 220 μm in the main channel were all consistently higher in the stable gradient created by the conditioned medium from LPS-activated mature DC throughout the 40-minute analysis (Figure 2D), when compared to that of the plain medium control.

### 4.2. Induced Cell Migration Lengthens NK–DC Interaction Time in D^3^-Chip

**Figure 3:**
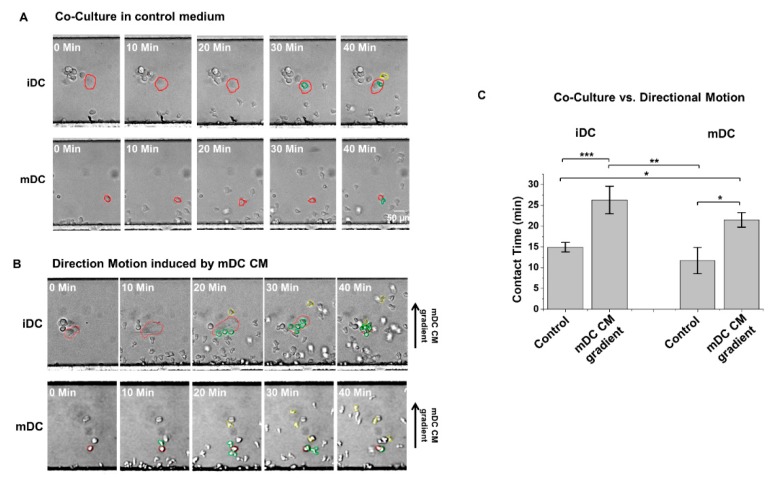
Induced cell migration lengthened NK–DC interaction time observed in the D^3^-Chip device. Representative cell images of NK–DC interactions recorded at specific time points (0, 10, 20, 30 and 40 min) in which (**A**) activated NK cells were not directed toward the loaded iDC (immature DC), mDC (LPS-activated mature DC) by using a plain complete tissue culture “control” medium in a “co-culture” setting; (**B**) activated NK cells were promoted to migrate toward the loaded iDC, mDC by the use of the gradient generated from the mDC CM (conditioned medium of mDC). Images were corrected on PowerPoint to articulate cell outlines for clear presentation. DCs outlined in red are cells of interest, NK cells outlined in green are cells currently interacting with a DC at the given time frame, and NK cells outlined in yellow are cells that previously interacted with the DC of interest. (**C**) Average contact time (in minutes) of the observed NK–DC interactions in iDC and mDC. The error bar indicates the standard error of the mean (SEM). *, **, and *** indicate *p* < 0.05, *p* < 0.01, and *p* < 0.001, respectively, using the one-way ANOVA test.

NK–DC interactions can be contact-dependent as well as contact-independent [19]. We, therefore, first attempted to use the D^3^-Chip devices we described above to examine quantitative differences, if any, in the activated NK-cell interactions with either immature or mature DC in vitro. Activated NK cells were seeded through the cell loading inlet to align at the docking structure relative to the concentration gradient. Concurrently, either immature DC or LPS-mature BM-derived DCs were seeded and attached randomly throughout the main gradient generating channel (Figure 3). To distinguish NK cells from DC, we used either DC from C57BL/6 and EYFP + NK cells isolated from the CAG-EYGP transgenic mice or NK from C57BL/6 and EYFP + DC cells derived from the bone marrow of the CAG-EYGP transgenic mice in these NK–DC studies. The uses of EYFP + NK/C57BL/6 DC or C57BL/6 NK/EYFP + DC in these studies did not show any differences in the NK-migratory responses we analyzed here (Figure 2, Supplementary Figure S1; Supplementary Movies S1 and S4). Seen in this design, interactions of NK and DC required migration of the docked NK cells to find a loaded DC target in the chamber of the microfluidic device. As we demonstrated that conditioned medium from the LPS-mature BM-derived DC provided a strong chemotactic signal to NK cells (Figure 2) [15], we, therefore, used the conditioned medium from the LPS-mature BM-derived DC to create a stable chemotactic gradient in the device to promote NK-cell migration.

We observed that very few NK cells had come into contact with the loaded DC when we used a plain, complete tissue culture medium in the devices under a “co-culture” condition (Figure 3A, Supplementary movie S2). Nevertheless, contact time between a NK cell and a DC (either immature or mature) could be measured. To contrast, the use of the conditioned medium from LPS-activated DC promoted NK-cell migration toward the loaded DC (as shown in the “mDC CM” condition in Figure 3B) in the devices and increased the number of NK cells making contacts with the loaded DC (Figure 3, Supplementary movie S2). Multiple NK cells were found to simultaneously come into contact with an immature DC (Figure 3, Supplementary movie S2). When we measured the contact time between NK–DC under this “directional” condition, we observed that chemotaxis (driven by the chemical gradient created by the conditioned medium from LPS-activated DC) facilitated significantly longer interaction times between NK cells and DC, regardless of the activation states of the DC (immature versus mature), when compared to that observed under the “co-culture” condition (Figure 3C). We did not observe any significant differences between the contact time of NK-immature DC or NK-mature DC under either “co-culture” or “directional” conditions (Figure 3C).

### 4.3. The Use of a Novel T^2^-Chip to Further Examine NK–DC Interactions

**Figure 4:**
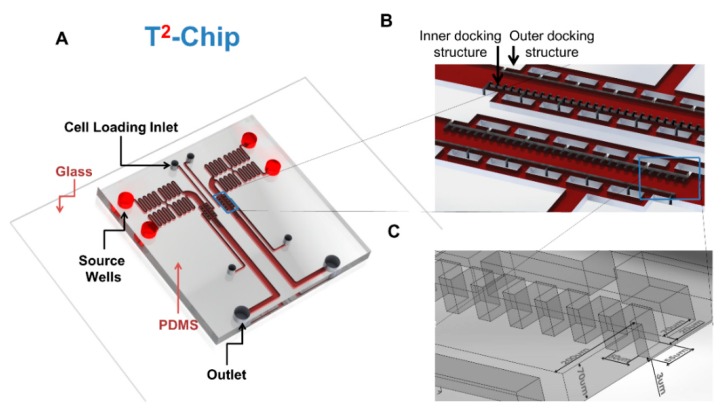
Schematic and representation of the T^2^-Chip device. (**A**) A schematic illustration of the two-unit T^2^-Chip device, indicating the locations of the source wells, outlets and cell loading entrances; (**B**) A close-up view of the pair of docking structures indicates the position of the compartment-like inner docking structure and traditional outer docking structure. (**C**) A detailed depiction of the dimensions for the two docking structures.

**Figure 5:**
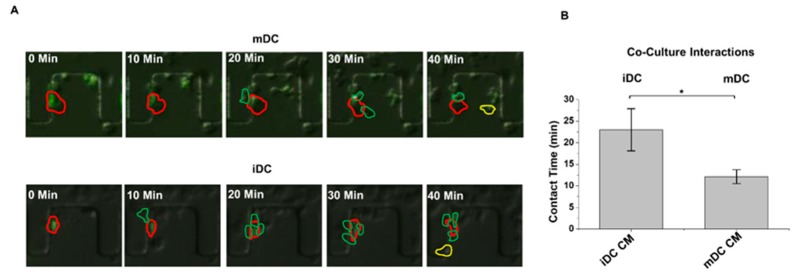
Contact times of the activated NK cells and EYFP + DCs in the T^2^-Chip device. (**A**) Representative cell images of co-culture experiments of NK-immature DC and NK-mature DC interaction experiments in the T^2^-Chip device, at time 0, 10, 20, 30, and 40 min. DCs outlined in red were cells of interest; DCs outlined in yellow were cells that had previously interacted with an NK cell, and NK cells outlined in green were cells that were currently interacting with a DC. (**B**) Duration of cell-interaction contact time between NK-immature DC and NK-mature DC. The error bar indicates the standard error of the mean (SEM). *, **, and *** indicates *p* < 0.05, *p* < 0.01, and *p* < 0.001, respectively, using the one-way ANOVA test.

**Figure 6:**
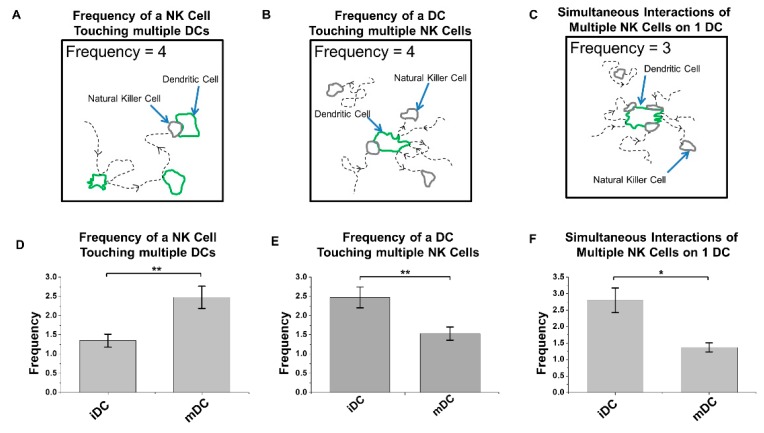
Frequencies of the activated NK cell and EYFP + DC interactions in the T^2^-Chip device. Cartoon schematic defining (**A**) frequency of NK cells touching DCs, (**B**) frequency of DCs touching NK cells, and (**C**) cell count of NK–DC simultaneous interaction. Live-cell assessment of (**D**) the frequency of which an NK cell came into contact with a DC, (**E**) frequency of which a DC came into contact with an NK cell, and (**F**) cell count of the highest number of simultaneous NK–DC interaction within the 40 min time span. The error bar indicates the standard error of the mean (SEM). *, **, and *** indicates *p* < 0.05, *p* < 0.01, and *p* < 0.001, respectively, using the one-way ANOVA test.

We created a new device (T^2^-Chip) to further facilitate analyses of NK–DC cell–cell interactions without the need for promoting NK-cell migration a long distance to find a loaded DC. The latter will support a higher frequency of NK–DC cell–cell interactions to be analyzed under defined conditions in vitro. Using this T^2^-Chip device, we ”trapped” (Figure 4) one cell type in small compartments of the inner side of the channel first, while allowing a second cell type to dock through the cell loading inlet and align cells relative to the trapped cells (Figure 4A), similar to in D^3^-Chip devices. Each compartment acted as an individual micro-environmental chamber that housed a range of cells depending on cell loading accuracy, which were used as test sites for NK–DC communication (Figure 4B). The entire docking barrier, including the trap compartments, had a ~3μm gap so cells could freely explore other compartments if they wished by changing their morphology and squeezing under the trap structures (Figure 4C). Similar to the D^3^-Chip, the T^2^-Chip was a Y-shaped device variant with a pressure balance segment to generate stable chemical gradients without the use of external pumps. FITC-dextran was applied to establish a gradient in this device, which performed similar to the D^3^-Chip, allowing for stable gradients for up to an hour. Unlike the D^3^-Chip, the T^2^-Chip allowed for better-controlled seeding of two different cell types (e.g., immature or mature DCs and IL-2 pre-treated NK cells) into multiple defined cell compartments within the channel. Within each compartment of the device, physical interactions between two cell types could be assayed in real-time at multi-cells level. We loaded IL-2 activated NK and DC (immature or mature) cells from the CAG-EYFP transgenic mice into the T^2^-Chip devices, in the presence of the conditioned medium from immature or mature DC, respectively, to study NK–DC contacts. At each time interval (0, 10, 20, 30 and 40 min), we tracked the NK and DC that came into contact in this T^2^-Chip. We determined the contact time these cells made in vitro. We observed that activated NK cells made contact with either mature DC (Figure 5A, top panel; Supplementary movie S3) or immature DC (Figure 5A, lower panel; Supplementary movie S3). However, the contact time between the activated NK cells and immature DC was significantly longer than that of the mature DC (Figure 5B). Additionally, our live-cell imaging allowed us to also enumerate the frequency of an NK cell touching multiple DCs, the frequency of a DC touching multiple NK cells or simultaneous interactions of multiple NK cells on a single DC (immature versus LPS-mature) over time (Figure 6). We were able to determine frequency of a NK cell touching multiple DCs (Figure 6A), frequency of a DC touching multiple NK cells (Figure 6B) and also simultaneous interactions of multiple NK cells on one DC (Figure 6C). Interestingly, we observed that NK cells had a significantly higher frequency in coming into contact with the mature DCs than immature DCs (Figure 6A). In contrast, there was a significantly higher frequency of an immature DC coming into contact with multiple NK cells (Figure 6B), and/or making multiple simultaneous contacts with multiple NK cells (Figure 6C).

## 5. Discussion

We reported here novel applications of two microfluidic devices (D^3^-Chip, the T^2^-Chip) to advance high throughput analyses of NK-cell migration properties, and also quantitation of duration and frequency of cellular contacts in NK–DC cell–cell interactions in vitro. Using these microfluidic devices, we measured NK-cell chemotaxis, migration speed and migration distances under specific chemical gradients (Figure 2). We examined NK–DC interactions with (Figure 3) or without the need for cell migration over a “long” distance (Figure 5 and Figure 6). We observed that NK cells had a significantly higher frequency in coming into contact with the mature DCs than immature DCs (Figure 6A). To contrast, there was a significantly higher frequency of an immature DC coming into contact with multiple NK cells (Figure 6B), and/or making multiple simultaneous contacts with multiple NK cells (Figure 6C).

The importance of NK–DC crosstalk in regulating immune responses in infections and immunotherapy has been well established [12,20,21]. Advances in intravital imaging of T cells and NK cells in lymph nodes revealed interesting dynamic migration behaviors that involve structural and chemical cues in promoting effective cell–cell interactions in vivo [22,23]. Unlike an antigen-specific CD8 + T cell that will form stable DC interactions upon encountering an antigen-presenting DC [22], NK cells maintained motility during their activation mediated by either adoptive transfer of DCs or by injection of poly I:C or LPS in vivo [24,25]. Short and transient interactions with DC is sufficient to induce progressive NK-cell activation and subsequent enhancement of its effector functions [23]. Regarding the bi-directional NK–DC interactions, however, activated DC could induce NK-cell activation via contact-dependent and/or soluble signals, NK-cell proliferation via cytokines (such as IL-12, IL-18) [23]. Reciprocally, activated NK cells could induce either DC maturation via cytokines (such as IFN-γ and TNF), or DC lysis in cell-contact dependent interactions [26]. Given that multiple DC subsets exist, the maturation status of each DC subset could define different functional properties of the DC [19], and that NK cells could be either in a “resting” state or activated by different cytokines in vivo [27], we reasoned that an in vitro microfluidic platform could, in principle, provide us additional insights into the migration properties of NK cells under specific NK–DC experimental conditions. Here, we provided proof-of-concepts to the applications of two novel devices in our study of NK-DC crosstalks. Notably, we observed that mature DC promoted a significantly higher frequency of activated NK cell contacts when compared to that of the immature DC (Figure 6A). To contrast, immature DC promoted a significantly higher frequency of multiple NK-cell contacts (Figure 6B), and multiple simultaneous contacts with multiple NK cells (Figure 6C). Such differences in the contact times and number of contacts made between the “scanning” NK cells and DC (immature versus mature) may be determined by the overall balance of the activation and inhibitory signals integrated by the NK receptors upon engagements of their ligands on the immature and mature DC. Collectively, these differences in NK-DC interactions may underlie the differential maturation of immature DC by activated NK cells.

This work contributes to a growing niche of development of advanced microfluidic devices to better understand how various physical and environmental cues can stimulate and guide immune cell motility [28]. Further applications of these microfluidic devices in studying how NK cells activated by different cytokines differ in their abilities to respond and/or interact with specific DC subsets of different maturation states defined in vitro will allow us to delineate further the functional regulations of NK–DC crosstalk, and its subsequent cellular responses, under different microenvironments.

## Supplementary Materials

The following are available online at https://www.mdpi.com/2072-666X/10/12/851/s1, Figure S1: EYFP+ C57BL/6 NK cell migration assessed in the D^3^-Chip; Video S1: Supplementary Movie 1 for Figure 2. Migration of NK cells in a D^3^-Chip device in a control medium and in a gradient of mature dendritic cell supernatant; Video S2: Supplementary Movie 2 for Figure 3. Directional migration interaction of NK cells with immature dendritic cells and mature dendritic cells in a control and in a gradient of mature dendritic cell supernatant in a D^3^-Chip device.; Video S3: Supplementary Movie 3 for Figure 5. Interaction experiment between NK cells with immature dendritic cells, and NK cells with mature dendritic cells in a T^2^-Chip device.; Video S4: Supplementary Movie 4 for Supplementary Figure S1. Migration of EYFP+ NK cells in a D^3^-Chip device in a control medium and in a gradient of mature dendritic cell supernatant.
