# Supplementary material for: A New Microfluidic Platform for Studying Natural Killer Cell and Dendritic Cell Interactions
Source: Micromachines (Basel). 2019 Dec 5;10(12):851. doi: 10.3390/mi10120851 (PMC6952968; doi:10.3390/mi10120851)
Supplement: Supplementary file 1 [file micromachines-10-00851-s001.zip › 3SFigureBinder120419/3Supplementary figures legends_JH.DOCX]

**Supplementary figure legends:**

**S1. EYFP+ C57BL/6 NK cell migration assessed in the D^3^-Chip.** (A) Representative cell images of NK cell chemotaxis in a culture medium control, mature DC CM gradient at time 0, 10, 20, 30, and 40 minutes. Images were corrected on PowerPoint to articulate cell outlines for clear presentation. (B) Cell speed and (C) migration distance for the culture medium control, mature DC supernatant gradient and immature DC supernatant gradient at time 0, 10, 20, 30, and 40 minutes. The error bar indicates the standard error of the mean (SEM). *, **, and *** indicates *p*<0.05, *p*<0.01, and *p*<0.001, respectively, using the one-way ANOVA test. (D) A histogram representing an NK cell count distribution at different distances of the D^3^-Chip at time 10, 20, 30 and 40 minutes. Cell counts were organized into six sections from 0-36μm, 37-73μm, 74-109μm, 110-146μm, 147-183μm, and 184-220μm.

**Supplementary Movie 1 for Figure 2.** Migration of NK cells in a D^3^-Chip device in a control medium and in a gradient of mature dendritic cell supernatant. Time lapse of NK cells were taken for 40 minutes.

**Supplementary Movie 2 for Figure 3.** Directional migration interaction of NK cells with immature dendritic cells and mature dendritic cells in a control and in a gradient of mature dendritic cell supernatant. Induced migration interaction experiments were done in the D^3^-Chip Device for 40 minutes.

**Supplementary Movie 3 for Figure 5**. Interaction experiment between NK cells with immature dendritic cells, and NK cells with mature dendritic cells. Interactions were monitored in the compartments of the T^2^-Chip Device for 40 minutes.

**Supplementary Movie 4 for Supplementary Figure 1**. Migration of EYFP^+^ NK cells in a D^3^-Chip device in a control medium and in a gradient of mature dendritic cell supernatant. Time lapse of NK cells were taken for 40 minutes.
