# Supplementary figures and images for: A New Microfluidic Platform for Studying Natural Killer Cell and Dendritic Cell Interactions
Source: Micromachines (Basel). 2019 Dec 5;10(12):851. doi: 10.3390/mi10120851 (PMC6952968; doi:10.3390/mi10120851)

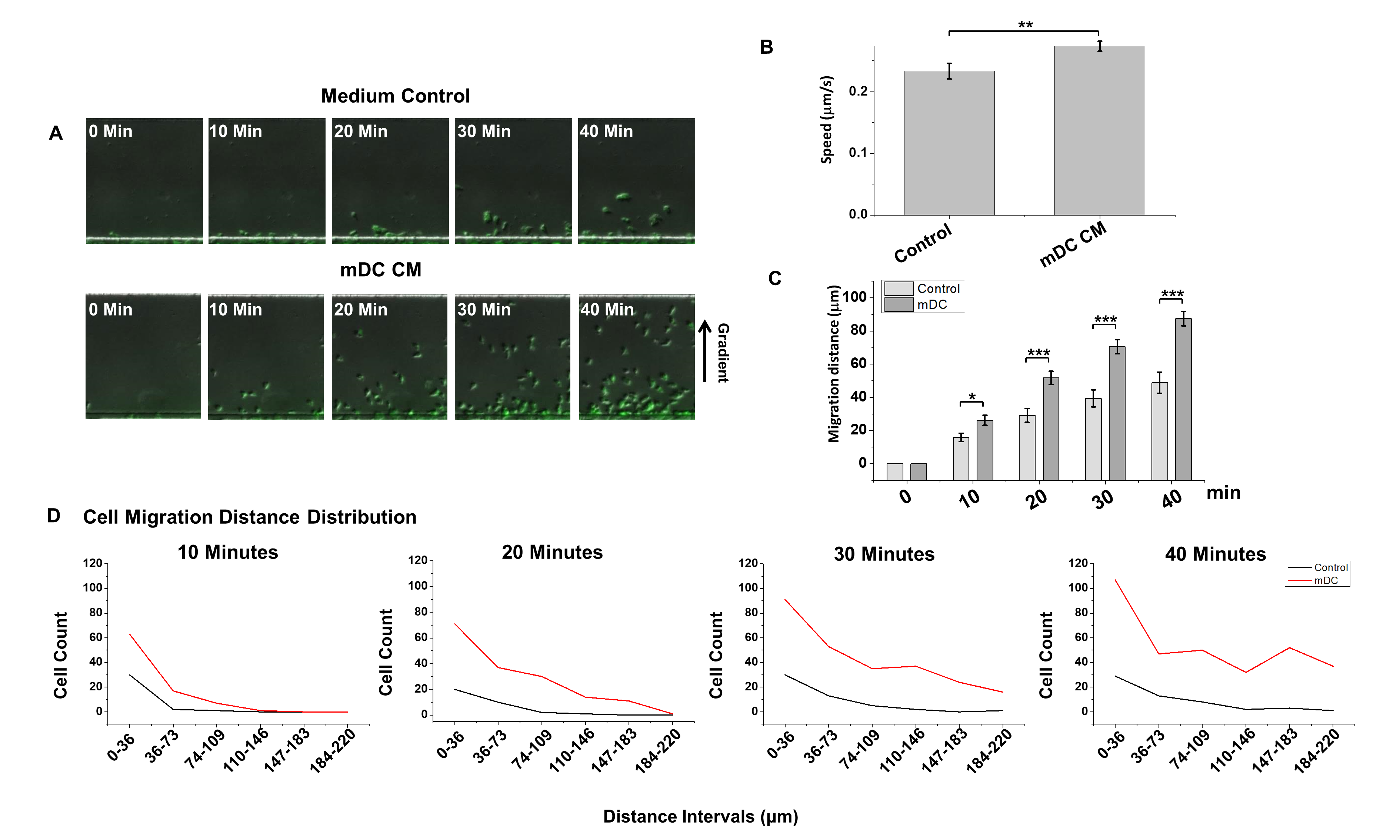

Supplement: Supplementary file 1 [file micromachines-10-00851-s001.zip › 3SFigureBinder120419/SFig 1.TIF]
